# Evaluation and Management of Angioedema in the Emergency Department

**DOI:** 10.5811/westjem.2019.5.42650

**Published:** 2019-07-02

**Authors:** Brit Jeffrey Long, Alex Koyfman, Michael Gottlieb

**Affiliations:** * Brooke Army Medical Center, Department of Emergency Medicine, Fort Sam Houston, Texas; † The University of Texas Southwestern Medical Center, Department of Emergency Medicine, Dallas, Texas; ‡ Rush University Medical Center, Department of Emergency Medicine, Chicago, Illinois

## Abstract

Angioedema is defined by non-dependent, non-pitting edema that affects several different sites and is potentially life-threatening due to laryngeal edema. This narrative review provides emergency physicians with a focused overview of the evaluation and management of angioedema. Two primary forms include histamine-mediated and bradykinin-mediated angioedema. Histamine-mediated forms present similarly to anaphylaxis, while bradykinin-mediated angioedema presents with greater face and oropharyngeal involvement and higher risk of progression. Initial evaluation and management should focus on evaluation of the airway, followed by obtaining relevant historical features, including family history, medications, and prior episodes. Histamine-mediated angioedema should be treated with epinephrine intramuscularly, antihistaminergic medications, and steroids. These medications are not effective for bradykinin-mediated forms. Other medications include C1-INH protein replacement, kallikrein inhibitor, and bradykinin receptor antagonists. Evidence is controversial concerning the efficacy of these medications in an acute episode, and airway management is the most important intervention when indicated. Airway intervention may require fiberoptic or video laryngoscopy, with preparation for cricothyrotomy. Disposition is dependent on patient’s airway and respiratory status, as well as the sites involved.

## INTRODUCTION

Angioedema is a condition defined by non-dependent, non-pitting, transient edema lasting up to seven days due to the accumulation of vasoactive substances.^1^–^5^ These substances increase vascular permeability, resulting in swelling in the deep dermal, submucosal, and subcutaneous tissues of the face, lips, neck, extremities, and gastrointestinal (GI) system.^1^,^2^,^6^–^9^ Urticaria may be present in up to 50% of cases, depending on the underlying process.^1^,^2^,^6^–^9^

Angioedema accounts for 80,000 to 112,000 emergency department (ED) visits per year, with a hospitalization rate of 4.0 per 100,000 population.^10^–^12^ For patients taking angiotensin-converting enzyme inhibitors (ACEi), the incidence of angioedema ranges from 0.1–0.7% over a patient’s lifetime, while the prevalence of hereditary angioedema (HAE) ranges from 1 in 10,000 to 1 in 50,000 persons.^6^,^10^–^15^ Over 50% of patients with HAE require ED management, with over half of patients admitted to the hospital.^1^–^3^ ACEi-mediated angioedema accounts for 30% of angioedema cases. Of the cases of ACEi-mediated angioedema, one study found 18% of patients were admitted to observation, 12% to the inpatient setting, and 11% to the intensive care unit.^1^,^16^ Due to risk of airway involvement and death, the emergency physician (EP) plays a key role in assessment and management of angioedema.^1^,^2^,^17^,^18^

## METHODS

We searched PubMed and Google Scholar for articles in English from 1966 to October 2018 using a combination of the keyword and medical subject heading “angioedema” for production of this narrative review. Our search included case reports and series, retrospective and prospective studies, systematic reviews and meta-analyses, narrative reviews, and clinical guidelines. Two authors decided by consensus which studies to include for the review. Initial literature search revealed over 500 articles, of which 185 were selected for inclusion, focusing on ED evaluation and management.

## DISCUSSION

### Etiology

Angioedema can be defined as either hereditary (bradykinin) or acquired (bradykinin or histamine) (Table 1).^1^–^5^,^7^,^15^,^19^–^24^ The underlying pathophysiology (ie, bradykinin-vs histamine-mediated) influences the clinical presentation and treatment recommendations.^1^,^2^,^7^,^8^ Bradykinin-mediated forms are generally more severe, longer lasting, and frequently involve the upper airway and gastrointestinal (GI) system.^1^,^20^–^24^

### Histamine-mediated

Histamine-mediated angioedema is the most common form, accounting for 40–70% of all cases, and is associated with immunoglobulin E resulting in degranulation of mast cells and basophils.^1^–^5^ H1 and H2 receptors are primarily responsible for the swelling that leads to angioedema.^1^,^4^,^5^ Histamine-mediated angioedema, such as anaphylaxis, occurs rapidly after an allergen exposure (type I hypersensitivity reaction).^5^,^25^–^30^ Histamine-mediated angioedema and anaphylaxis present similarly, as they are along the same clinical spectrum, although diagnosis of anaphylaxis requires specific clinical criteria.^1^–^5^ Importantly, therapy for histamine-mediated angioedema and anaphylaxis is the same, which will be discussed later. Histamine-mediated angioedema typically resolves within 24–48 hours. This form can result from food allergens, medications, exercise, bites, stings, or latex exposure.^31^–^33^ There is also a form of physically-induced angioedema from cold exposure, heat pressure, physical activity, ultraviolet radiation, and vibration, which is most likely due to histamine release.^34^–^36^

**Population Health Research Capsule**

What do we already know about this issue?

Angioedema is defined by non-dependent, non-pitting edema that affects several different sites and is potentially life-threatening due to laryngeal edema.

What was the research question?

This narrative review evaluates the pathophysiology, evaluation, and management of angioedema.

What was the major finding of the study?

There are two forms of angioedema. Management must focus on the airway, although several medications are promising.

How does this improve population health?

Evidence is controversial for the efficacy of several medications, and airway management is the most vital intervention if indicated. Disposition depends upon airway and respiratory status.

### Bradykinin-mediated

Bradykinin-mediated pathways involve this vasoactive nonapeptide that activates endothelial cells.^5^,^14^,^37^ Several systems regulate bradykinin, including the coagulation, complement, and contact pathways.^4^,^38^ Excess bradykinin is due to production, release, or inhibition of its breakdown.^39^–^41^ This form comprises drug-induced angioedema (ie, ACEi-mediated), HAE types I and II, and several forms of acquired and idiopathic angioedema.^13^,^20^,^42^,^43^

ACEi-mediated angioedema accounts for up to 30% of ED visits for angioedema of all types.^16^,^21^,^44^–^49^ ACEis prevent the conversion of angiotensin I to angiotensin II and reduce bradykinin metabolism, which increases the risk of angioedema. Most cases are localized to the lips and tongue.^14^,^15^ Patients at greatest risk for developing ACEi-mediated angioedema include African Americans and those taking immunosuppressants or dipeptidyl peptidase-IV inhibitors (a class of diabetic medications) in addition to the ACEi.^14^,^15^,^49^ The rate of angioedema is highest within the first 30 days of starting an ACEi, although the risk of angioedema remains for the duration of the ACEi use, with cases of ACEi-mediated angioedema documented in patients with prolonged courses of multiple years.^50^–^52^ If a patient continues taking an ACEi after developing ACEi-mediated angioedema, the average time to recurrence is approximately 10 months.^50^,^53^ Angiotensin II receptor blockers (ARB) and renin antagonists can also cause angioedema, but this is not due to bradykinin.^1^–^5^ If angioedema develops in a patient on an ACEi, the ACEi should be discontinued and a different antihypertensive class used.

HAE is thought to be autosomal dominant with abnormal C1-INH amounts and/or function. HAE affects approximately 1 in 10,000–50,000 people.^1^,^2^,^54^,^55^ Type 1 is due to decreased and defective C1-INH and is the most common (85%) form of HAE, followed by type II which is caused by dysfunctional C1-INH.^13^,^55^–^59^ A third form of HAE with normal C1-INH has also been described.^60^–^62^ Most patients present by age 10 with recurrent episodes of edema.^1^–^4^,^58^ HAE is often associated with prodromal symptoms, such as erythema marginatum, but not urticaria.^1^,^4^,^16^,^61^,^63^ HAE occurs more commonly in females and causes more severe swelling with significant face and tongue involvement when compared with males.^1^–^5^,^13^ Estrogen-containing medications and pregnancy increase the attack frequency in female patients.^7^,^61^,^64^

Acquired angioedema appears similar to HAE with C1-INH deficiency, but this is not hereditary and more commonly affects those > 40 years.^1^,^65^–^71^ This form is most commonly due to catabolism of C1-INH, although some patients may have a lymphoproliferative or autoimmune disorder.^1^,^65^–^71^

A less common cause of non-histaminergic angioedema is associated with medications, including nonsteroidal anti-inflammatory drugs (NSAID), antibiotics, and ARB.^72^–^75^ NSAID-associated angioedema results from inhibition of cyclooxygenase and accumulation of leukotriene mediators, and occurs in 0.1–0.3% of patients taking an NSAID.^56^ Exposure to recombinant tissue plasminogen activator therapy in acute ischemic stroke is also associated with angioedema, occurring in 1.2–5.1% of patients, with increased risk in patients taking an ACEi.^76^–^80^ Most of these cases are mild and resolve in 24 hours.^1^,^2^,^7^,^8^

### Idiopathic

Idiopathic angioedema is diagnosed by failure to determine the etiology with ≥ 3 attacks in a 6–12 month period.^2^,^5^,^81^–^83^ Most patients with idiopathic angioedema will demonstrate a response to standard therapies for anaphylaxis (eg, epinephrine, antihistamines, steroids), although a small group will not improve with these therapies.^1^–^4^,^84^ This latter group is more commonly bradykinin-associated.^1^–^4^,^84^

### Presentation, History, and Physical Examination

Initial evaluation requires assessing vital signs, airway, and cardiovascular systems. Asphyxiation is the leading cause of mortality in these patients, necessitating airway evaluation.^17^,^18^,^85^ At least one episode of laryngeal edema occurs in over half of all patients with HAE and accounts for over 30% of deaths in HAE.^17^,^18^ Emergency physicians (EP) must inquire about lip swelling, tongue swelling, and GI symptoms (nausea, vomiting, diarrhea, and pain). Additional information to gather includes prior personal or family history of angioedema, medications, and related symptoms (eg, pruritis, dyspnea, syncope, lightheadedness).^85^–^93^ Patients with a known history of HAE should also be asked about recent trauma, which can trigger an episode.^1^–^5^ Most patients with HAE report prodromal symptoms prior to swelling, such as fatigue and rash.^94^

The presentation can vary depending upon the subtype but is primarily dependent upon whether the etiology is histaminergic or non-histaminergic (Table 2).^1^–^5^,^13^ The most commonly involved areas include the head and neck (eg, eyelids, lips, tongue, larynx), extremities (eg, hands and feet), external urogenital system, and abdomen.^1^–^5^,^9^,^13^,^28^ However, involvement of these sites is often non-contiguous, with no specific pattern.^1^–^5^,^7^,^8^ Histaminergic forms display faster onset, while HAE and acquired forms have a slower, progressive onset occurring over several hours.^1^,^2^,^7^,^8^ GI tract submucosal involvement occurs in up to 93% of patients with HAE and can cause symptoms that mimic bowel obstruction.^13^,^55^,^90^,^91^ Non-pitting edema is present in both histaminergic and non-histaminergic forms.^1^,^4^,^7^,^8^ Pruritic, localized, urticarial lesions may be present in histamine-mediated forms with involvement of the deep dermis, but these are rare in non-histaminergic forms.^13^,^95^,^96^ Urticaria occurs in approximately 50% of patients with histamine-mediated angioedema.^13^,^95^,^96^

Findings suggestive of the need for a definitive airway include stridor, hoarseness, dyspnea, and voice changes.^86^–^92^ The patient should be asked to phonate “E” with a high pitch, as a patient able to complete this maneuver is unlikely to have laryngeal edema.^1^,^7^,^8^ Auscultation of the lungs to determine the presence of wheezing is recommended.

Differentiating histamine and bradykinin-mediated angioedema can be difficult. One retrospective study evaluated 188 patients, with one point assigned to age > 65 years, dyspnea, no itching or erythema, laryngeal involvement, and intake of ACEi/AT-II antagonist, and two points assigned if there was no response to steroid therapy.^97^ If the score was ≥ 3 points, the patient was treated with C1-INH or B2 receptor antagonist for suspicion of bradykinin-mediated angioedema. This resulted in a sensitivity of 96% and specificity of 84% for the diagnosis of bradykinin-mediated angioedema.^97^ While this tool can help to differentiate the underlying etiology, it requires further validation before routine use.

### Diagnostic Testing

Angioedema is a clinical diagnosis, with no required testing in the ED.^1^,^2^,^4^ Leukocyte counts cannot reliably differentiate if an infection is present, as leukocytosis over 30,000 per cubic millimeter has been observed.^98^ C-reactive protein may be elevated in ACEi-mediated angioedema.^1^,^4^ Determining the specific type of angioedema involves specialized laboratory testing not available in the ED, including tryptase, C4, and C1-INH.^1^–^4^ These tests can be obtained in the outpatient setting and should not be routinely obtained in the ED, as they do not guide management. Histamine-mediated forms can display elevated tryptase levels during attacks, while patients with HAE will display normal tryptase levels.^3^ C4 levels serve as a sensitive screening test for C1-INH deficiency.^1^,^3^ Serum C4 levels will typically be < 30% of normal in acute episodes of angioedema from HAE types I and II, although the laboratory values may be normal between attacks.^23^,^100^,^101^ Type I HAE often involves low C1-INH levels and decreased function, while type II HAE includes normal levels but decreased function.^13^,^19^,^102^ C1q levels, a component of the complement system, can be used to differentiate acquired and hereditary forms, as C1q is decreased in acquired angioedema and normal in HAE.^1^,^3^,^4^,^100^,^103^ Type III HAE has normal levels and function of C1-INH but is usually identified by a positive family history.^1^–^4^,^7^,^8^ No tests can confirm ACEi-mediated angioedema.^1^,^7^,^19^,^23^

Patients with abdominal symptoms may demonstrate segmental bowel wall edema, straightening of intestinal segments, and ascites on computed tomography (CT).^4^,^104^,^105^ Ultrasound may similarly reveal bowel wall thickening or ascites.^106^ Ultrasound can be used to evaluate for laryngeal edema, although this requires further study.^4^ Chest radiography, if obtained, is typically normal. Neck radiographs and CT of the neck with intravenous (IV) contrast can evaluate for mimics of angioedema, but they should not be ordered routinely for patients with suspected angioedema.^104^ Fiberoptic visualization of laryngeal and airway structures is recommended if concern for laryngeal or airway involvement is present.

### Management

**Figure 1:**
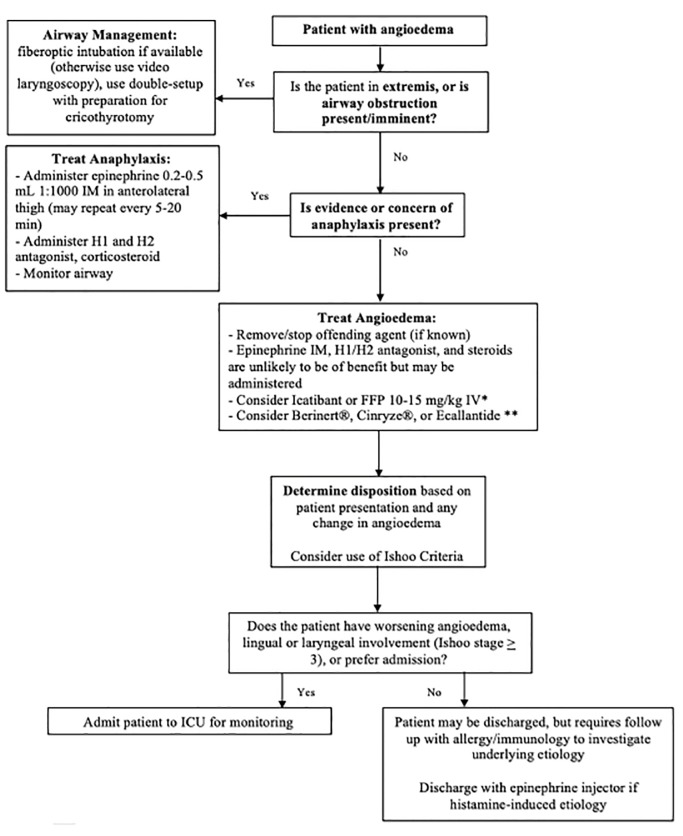
Algorithm for angioedema management. *IV*, intravenous; *IM,* intramuscular; *ACEi*, angiotensin converting enzyme inhibitor;* FFP*, fresh frozen plasma; *ICU,* intensive care unit. *ACEi-mediated, Hereditary, or Acquired Angioedma only. **Hereditary or Acquired Angioedema only.

The primary focus of ED management is assessment of the airway and evaluation for anaphylaxis, which is the most common mimic.^1^–^4^,^7^,^8^ Figure 1 depicts an algorithm for management. Vital signs should not be relied upon in isolation to determine the need for airway intervention.

#### Airway Management

Patients with angioedema involving the tongue or larynx require consideration of definitive airway management. Angioedema can progress rapidly within hours, and airway obstruction occurs in up to 15% of patients with angioedema.^1^,^4^,^17^,^18^ For patients with angioedema who require a definitive airway, cricothyrotomy or tracheostomy is needed in up to 50% of cases.^17^,^87^,^105^ Prior history of intubation or severe angioedema should raise the concern for a difficult airway which may require early airway intervention.^1^,^4^,^107^ Evidence of upper airway involvement on examination includes stridor, change in patient voice, and hoarseness. If physical examination reveals swelling of the tongue, floor of the mouth, or soft palate, directly visualize the tongue base and airway with fiberoptics. The presence of epiglottic, aryepiglottic, or laryngeal edema suggests need for definitive airway.^1^,^2^ If the angioedema exclusively involves structures anterior to the teeth such as the lips, intubation is generally not needed.^85^–^92^

Noninvasive positive pressure ventilation can also assist with temporization; however, this is not a definitive therapy for patients with airway involvement. Supraglottic and extraglottic airway devices are common rescue devices; however, they are not recommended in patients with angioedema, as the device will remain above the site of airway obstruction.^1^,^4^,^7^,^8^,^85^ If placed, these devices may also worsen edema due to the associated trauma with placement.

Physical manipulation of the airway may worsen edema, especially in bradykinin-mediated angioedema.^1^,^4^,^7^,^8^ In patients with history or evidence on examination of a difficult airway, video laryngoscopy or fiberoptic awake intubation is recommended, as this allows the patient to maintain his/her airway reflexes during airway visualization and the intubation attempt.^1^,^4^,^107^–^109^ Topical anesthetics and ketamine are optimal agents for awake intubation. Severe edema may prohibit passage of an endotracheal tube through the glottis, even with the use of fiberoptic or video laryngoscopy guidance. Thus, the resuscitation team must prepare for cricothyrotomy before an attempt at intubation is started, known as a double setup.^1^–^5^ If the patient does not require immediate airway intervention, transfer to the operating room may be beneficial with anesthesia and otolaryngology consultation, similar to pediatric epiglottitis.

#### Medications

Medication management focuses on three aspects: acute episode management, short-term prophylaxis, and long-term prophylaxis, with ED management focusing on the acute episode.^1^,^4^,^7^,^8^ If the suspected etiology is drug- or allergic-induced, the trigger must be removed.^1^–^5^ In histamine-mediated forms of angioedema, standard therapy for anaphylaxis is recommended. However, in other forms of angioedema including bradykinin-mediated forms, standard therapies for anaphylaxis should not be effective.^110^,^111^

Patients with evidence of histaminergic forms of angioedema and concern for airway involvement should receive epinephrine, steroids, antihistamines, and IV fluids.^1^–^5^,^7^,^8^,^112^ If there is any suspicion of anaphylaxis, urticaria-associated angioedema, or if the exact underlying cause of the angioedema is unknown, histamine-mediated edema should be assumed. Epinephrine should be administered via the intramuscular route into the anterolateral middle third of the thigh, with initial dose 0.3–0.5 milliliter (mL) (0.3–0.5 milligram [mg]) of 1:1000 dilution (1 mg/mL), which can be repeated every 5–20 minutes.^1^,^4^ Subcutaneous administration is not recommended.^112^–^114^ IV epinephrine should be considered in patients requiring multiple doses of intramuscular epinephrine and should begin at doses of 1–4 micrograms (mcg) per minute.^112^,^115^,^116^ Epinephrine can be administered peripherally by injecting 1 mg of epinephrine into 1 L of normal saline, resulting in a final concentration of 1 mcg/mL. If administered wide-open through an 18-gauge IV, this provides 20–30 mL/minute (20–30 mcg/minute) of epinephrine.

Adjunctive therapies for histamine-mediated angioedema include antihistamines and steroids.^1^–^5^,^7^,^8^,^112^ Antihistamines have a slower onset of action and should only be used as an adjunctive therapy.^1^–^5^,^7^,^8^,^112^,^117^ Diphenhydramine is an H1 antagonist that can be used in doses of 25–50 mg IV to reduce swelling in combination with a second- or third-generation antihistamine agent (eg, cetirizine, loratadine, fexofenadine, levocetirizine, desloratadine).^1^–^5^,^112^,^117^–^127^ The addition of an H2 antagonist is beneficial in decreasing urticaria, as 15% of cutaneous histamine receptors are H2.^117^–^128^ Steroids such as methylprednisolone 125 mg IV decrease inflammatory mediators in histamine-mediated angioedema and anaphylaxis but, similar to other medications, there is little to no evidence for their use in non-histaminergic angioedema.^1^,^4^,^7^,^8^,^112^ The onset of action after administration is delayed, typically requiring 4–6 hours to take effect.^1^–^5^,^7^,^8^,^14^,^15^

Fresh frozen plasma (FFP) has been recommended for use in angioedema based on case reports demonstrating improvement in HAE and ACEi-mediated angioedema,^129^–^133^ as FFP contains varying amounts of C1-INH.^1^–^4^,^7^,^8^,^14^,^15^,^134^–^136^ Several of the first case reports suggested FFP can be used as prophylaxis for HAE in patients undergoing dental procedures.^129^,^135^,^137^ A retrospective study suggests efficacy in decreased intubation frequency and intensive care unit (ICU) length of stay.^137^ However, type II HAE may worsen with FFP due to the presence of an autoantibody responsible for decreased C1-INH.^134^ Limited literature has described FFP in ACEi-mediated angioedema, primarily case reports and series demonstrating improved symptoms at four hours.^132^,^133^,^136^,^138^–^145^ Its use in acquired forms has not demonstrated efficacy, and a major limitation is the need to thaw FFP for use. The literature is inconsistent with regard to preferred dosing, with most studies giving 1–4 units (250–1000 cubic centimeters).^1^–^5^,^135^,^136^ FFP requires close to 50 times the volume of other medications with C1INH to obtain the same serum levels of enzyme.^135^ Risks include potential volume overload, transmission of bloodborne infection, and hypersensitivity reaction.^1^–^4^,^7^,^8^,^136^ FFP also contains substrates such as kallikrein and kininogen that may paradoxically worsen angioedema.^1^–^4^,^7^,^8^,^14^ Despite this theoretical effect, worsening of angioedema with FFP administration has not been found in cases of ACEi-mediated angioedema.^1^,^4^,^134^–^136^ There is no support for FFP in other acquired forms of angioedema.^1^–^4^,^135^

Prothrombin complex concentrate (PCC) has also been used for ACEi-mediated angioedema.^1^,^4^,^146^ However, the data is limited to one case report in which 1500 units were administered. Symptoms began to improve in 20 minutes, with resolution in eight hours.^146^ Four-factor PCC contains C1-INH, which may explain the improved symptoms.

### Targeted Therapies for Bradykinin-mediated Angioedema

Bradykinin-mediated forms of angioedema are typically resistant to therapies effective in histamine-mediated reactions.^1^,^4^,^7^,^8^,^13^,^135^,^136^,^147^ At the time of construction for this review, several medications have been FDA approved for treatment of acute bradykinin-mediated angioedema: three C1-INH concentrates (two plasma-derived and one recombinant), one kallikrein inhibitor, and one bradykinin-2-receptor antagonist (Table 3).^1^,^4^,^7^,^8^,^13^,^135^,^136^

#### C1 Inhibitor Concentrate

C1-INH concentrate for HAE episodes was first described in 1973, and there are two plasma-derived formulations currently available (Berinert and Cinryze), as well as one recombinant form (Ruconest), all administered intravenously.^1^–^5^,^7^,^8^,^135^,^136^,^148^ They are currently approved for acute HAE, although these medications have been used for ACEi-mediated forms.^1^–^5^,^7^,^8^,^135^,^136^,^148^ Berinert and Cinryze provide native plasma protein that regulates kallikrein and Factor XII activity, reducing bradykinin production. Bork et al. published a study based on 18 patients with 193 episodes of HAE, finding that the mean time to reversal was 42.2 minutes.^149^ The IMPACT trials (funded by CSL Behring, manufacturer of Berinert) evaluated Berinert vs placebo for acute episodes of HAE. IMPACT-1 found 20 units per kilogram (kg) improved time to symptom relief (0.5 hours vs 1.5 hours), but 10 units/kg did not.^150^,^151^ IMPACT-2 evaluated 1085 episodes of HAE in 57 patients, with a median time to symptom relief of 0.46 hours in patients receiving open-label Berinert.^151^ Cinryze has also been evaluated in HAE, with a double-blind placebo-controlled trial of 68 patients finding no statistically significant improvement in time to relief, although a double-blind crossover trial of 21 patients demonstrated decrease in attack number, duration, and severity.^4^,^136^,^152^ A study that was not placebo controlled found 68% of patients had improvement at one hour, while 87% experienced relief at four hours.^153^ Other trials evaluating Berinert and Cinryze for HAE and ACEi-mediated angioedema have found that the time to symptom improvement from administration varies from 0.5–5 hours, with complete resolution occurring within 1–10 hours.^136^ Ruconest is a recombinant form of C1INH. One open-label study with no placebo control found time to symptom relief of 30 minutes.^154^ A double-blind, placebo-controlled trial found time to symptom relief of 66 minutes in patients receiving 100 units/kg, vs 495 minutes in controls.^155^ Another randomized trial found time to symptom relief of 75 minutes in treated patients vs 303 minutes in patients receiving placebo.^156^

#### Kallikrein Inhibitor

Ecallantide (Kalbitor) is a recombinant plasma inhibitor of kallikrein provided subcutaneously and approved for use in HAE.^1^,^4^,^7^,^8^,^136^ This agent reduces bradykinin synthesis by preventing the cleavage of kininogen.^1^,^4^,^7^,^8^,^136^ It is associated with up to a 3% risk of anaphylaxis, necessitating close observation during administration and for up to one hour after.^1^,^4^,^7^,^8^ The EDEMA trials evaluated ecallantide for HAE.^157^–^160^ The EDEMA1 trial evaluated ecallantide at various doses vs placebo, finding the 40 mg/m^2^ dose improved symptoms at four hours, although other doses did not.^157^ The phase 2 EDEMA2 trial found subcutaneous dosing had improved outcomes vs IV dosing.^158^ EDEMA3 was an open label and double-blind phase 3 trial evaluating ecallantide vs placebo, with improvement in treatment score at four hours in patients receiving ecallantide.^159^ EDEMA4 found improved symptom scores vs placebo.^160^ A triple-blind phase 2 randomized controlled trial compared ecallantide at three different subcutaneous doses with placebo for ACEi-mediated angioedema and found no difference in patients meeting criteria for discharge.^161^ Lewis et al. conducted a double-blind phase 2 study with patients randomized to placebo or ecallantide.^162^ Most patients received therapy for histamine-mediated angioedema as well. The study found no difference in patients meeting criteria for discharge within six hours with ecallantide administration.^162^

#### Bradykinin B2 Receptor Antagonist

Icatibant acetate (Firazyr) is a selective and competitive bradykinin B2 receptor antagonist.^1^,^4^,^7^,^8^,^136^ Icatibant was evaluated in three clinical trials: FAST-1, FAST-2, and FAST-3.^136^,^163^,^164^ FAST-1 was a double-blind, placebo-controlled trial that demonstrated faster symptom relief (0.8 vs 16.9 hours) but no difference in the degree of symptom relief.^4^,^163^ The FAST-2 study demonstrated improved time to symptom relief in a double-blind study comparing icatibant to oral tranexamic acid (onset of symptom improvement 0.9 vs 7.8 hours).^4^,^163^ FAST-3 was a phase 3 double-blind, randomized, placebo-controlled trial that found a decrease in the time to primary symptom relief (2.0 vs 19.8 hours) and complete symptom relief (median 8.0 vs 36 hours).^4^,^164^ The literature suggests that the time to symptom improvement ranges from several minutes up to seven hours for icatibant. Of studies reporting improvement, approximately half of patients improve within 30 minutes, while time to complete symptom resolution ranges from 0.5–16 hours.^136^,^165^–^174^ Importantly, 40% of cases have complete resolution under four hours.^136^,^165^–^173^ A phase 2 multicenter, randomized controlled trial by Bas et al. evaluated icatibant vs steroids plus antihistamines, finding a shorter time to symptom relief with icatibant (2 hours vs 11.7 hours).^174^ More patients receiving icatibant also demonstrated resolution of edema at four hours. However, there was no difference in the degree of patient-reported symptom relief.^4^,^136^

A systematic review published in 2017 evaluating medication use in ACEi-mediated angioedema concluded icatibant possessed the highest level of evidence due to better study quality, while FFP has limited evidence demonstrating benefit and inconsistent dosing strategies for ACEi-mediated angioedema.^136^ This systematic review incorporated case reports, case series, a prospective observational study, and one randomized controlled trial. However, the recommendations were limited by low quality evidence and significant heterogeneity with respect to the severity of angioedema and clinical outcomes.^175^ Most of the included studies evaluated time to discharge and time to symptom relief, rather than the need for definitive airway, peak symptoms severity, duration of mechanical ventilation, and hospital/ICU admission.^136^,^175^ Publication bias was also severe, limiting conclusions. Studies following the publication of this systematic review from 2017 suggest no difference in time to discharge with icatibant.^175^ Sinert et al. evaluated icatibant vs placebo in a phase III, double-blind clinical trial.^176^ Time to discharge was four hours in both groups, with similar time of symptom relief.^176^ A second prospective, randomized study published by Straka et al. compared icatibant and placebo, finding no difference in symptom severity or duration.^177^

The current literature evaluating targeted therapy for bradykinin-mediated angioedema suffers from several limitations, including significant heterogeneity in patient selection, outcomes, comparators, dosing, and study design, as well as low numbers of included patients and high risk of bias.^1^,^4^,^136^,^175^ Medication efficacy is controversial with delayed onset of action, variable relief of symptoms, and limited availability depending on the institution.^136^,^175^ Rather than primarily focus on administering medications that may or may not improve symptoms in bradykinin-mediated forms of angioedema, EPs should focus on managing the patient’s airway.^1^,^4^,^175^

### Disposition

Disposition is mainly determined by airway involvement. Several studies have sought to predict airway compromise in patients with acute angioedema.^1^–^5^,^85^,^178^ Ishoo et al. performed a retrospective study of 80 patients with 93 acute episodes of angioedema.^85^ Wheezing, voice change, hoarseness, and stridor predicted the need for airway intervention. This study categorized patients based on the anatomic location of angioedema (Table 4).^85^ A subsequent study published one year later found the same factors predict need for definitive airway.^178^ Importantly, these factors require further validation and laryngeal visualization for staging.

Patients with respiratory or airway distress require ICU admission, as well as those with stage III and IV edema due to risk of progression.^1^–^5^,^7^,^8^,^89^ Patients with stable or improving stage I or II edema of the face, lip, or soft palate should be monitored for several hours to evaluate for worsening of the angioedema.^1^–^5^,^7^,^8^,^89^,^102^ Patients with stage I angioedema can be discharged with follow-up after evaluation for progression. Patients with stage II angioedema are often discharged home within 24 hours, and ED observation units provide an optimal setting for monitoring of these patients.^1^,^4^ However, if edema involves ≥ 3 sites (lips, tongue, mouth floor, soft palate, and larynx), admission is recommended due to greater risk of airway involvement.^89^

Patients with acute and recurrent angioedema may benefit from consultation with allergy/immunology specialists to discuss laboratory testing and arrange follow-up, particularly in patients with HAE.^1^–^5^,^7^,^8^,^102^ Patients with a first episode of angioedema, no response to anaphylaxis treatment, or family history of HAE require follow-up with an allergy/immunology specialist. These specialists can help diagnose a specific cause, evaluate and educate the patient concerning triggers, and provide prophylactic medications, which may prevent the need for ED care.^1^,^4^,^179^,^180^ Patients with known HAE and a recurrent attack may present with an action plan and recommended therapies, which should be followed when possible.^102^,^181^,^182^

Patients discharged from the ED with histamine-mediated angioedema and those with unclear etiology or first-time episode should be prescribed epinephrine autoinjectors and educated on potential triggers.^69^,^102^ Family and friends should also be educated on these factors. Patients with respiratory distress or airway swelling after discharge should use the epinephrine autoinjector and immediately return to the ED. The patient with ACEi-mediated angioedema must discontinue his or her medication, and an alternative agent should be discussed with the patient’s primary care provider.^1^–^5^,^7^,^8^,^102^ Most patients can use calcium channel blockers or angiotensin receptor blockers without developing a recurrence of their angioedema.^1^,^183^,^184^ The literature suggests the incidence of angioedema with ARB is 0.11%, which is not statistically different than placebo.^185^

## CONCLUSION

Angioedema is non-dependent, non-pitting edema at a variety of sites. Its forms can be divided into histamine-mediated and bradykinin-mediated types. Histamine-mediated forms can present similarly to anaphylaxis, while bradykinin-mediated angioedema is slower in onset, presents with greater face and oropharyngeal involvement, and has higher risk of progression. Initial evaluation and management should focus on the airway, followed by an evaluation for family history, medications, and prior episodes. Histamine-mediated angioedema is treated like anaphylaxis with epinephrine, antihistamines, and steroids. These medications are not effective for the bradykinin-mediated forms, although they can be attempted in the absence of effective therapy. Other medications include C1-INH protein replacement, kallikrein inhibitor, and bradykinin receptor antagonists. Several studies have evaluated these for angioedema, but the evidence is lacking for efficacy. The focus should be on airway management rather than medications in bradykinin-mediated angioedema. This may require fiberoptic or video laryngoscopy, with preparation for cricothyrotomy. Disposition depends on patient’s airway and respiratory status, as well as the involved sites.

## Figures and Tables

**Table 1:** Types of angioedema.

Types	Characteristics
Histamine-mediated (with urticaria)	- Allergy to food, venom, latex, medication - Acute or chronic spontaneous urticaria - Urticaria/angioedema associated with cold urticaria, vasculitis, exercise, episodic angioedema, vibration-induced, drug reaction
Bradykinin-mediated (without urticaria)	- Type I HAE: defective C1-INH level/function - Type II HAE: defective C1-INH function - Type III HAE: normal C1-INH - Acquired C1-INH deficiency: Type I associated with increased catabolism of C1-INH (lymphoproliferative disorder, autoimmune disease); Type II associated with autoantibody to C1-INH - ACEi-mediated angioedema - Medication associated: dipeptidyl peptidase-IV inhibitor (gliptins for diabetes mellitus), angiotensin II receptor blockers, recombinant tissue plasminogen activator, sirolimus, tacrolimus, everolimus
Idiopathic (unknown etiology)	- Histaminergic - Nonhistaminergic

*HAE*, hereditary angioedema; *C1-INH*, C1 inhibitor;* ACEi*, angiotensin-converting enzyme inhibitor.

**Table 2:** Comparison of features between non-histaminergic and histaminergic angioedema.

Features	Histaminergic	Non-histaminergic
Onset	Minutes	Hours
Duration	12–24 hours	48–72 hours
Hypotension	Common	Atypical
Urticaria	Common	Atypical
Bronchospasm; wheezing	Common	Atypical
Laryngeal edema	Possible	Possible
Abdominal pain	Possible	Possible
Therapy with epinephrine, antihistamines, steroids	Effective	Not effective

**Table 3:** Angioedema medications.

Medication (trade name)	Mechanism	Route	Dose	Time to onset	Minor side effects	Serious side effects
Plasma derived C1-INH (Berinert, Cinryze)	C1-INH protein replacement	IV	Berinert 20 units/kg; Cinryze 1000 units	Median 30–48 minutes	Dysgeusia	Hypersensitivity, thrombosis, blood-borne infection
Recombinant C1-INH (Ruconest)	C1-INH protein replacement	IV	50 units/kg	Median 90 minutes	Pruritis, rash, sinusitis	Hypersensitivity, anaphylaxis
Ecallantide (Kalbitor)	Kallikrein inhibitor	SQ	30 mg	Median 67 minutes	Headache, injection site reactions, nausea, fever	Hypersensitivity, anaphylaxis
Icatibant acetate (Firazyr)	Bradykinin B2 receptor antagonist	SQ	30 mg	Median 2 hours	Elevated LFTs, injection reaction, dizziness, headache, nausea, fever	Theoretical worsening of an ongoing ischemic event
Fresh frozen plasma	C1-INH protein replacement (various amounts)	IV	15 mg/kg	Minutes to hours		Hypersensitivity, worsening angioedema, transfusion infection

*C1-INH*, C1 inhibitor; *IV*, intravenous; *SQ*, subcutaneous; *LFTs*, liver function tests; *mg*, milligram; *kg*, kilogram.

**Table 4:** Predicting airway compromise based on anatomic location of angioedema.^85^

Stage	Site	Frequency	Discharge	Inpatient	ICU	Intervention
I	Face, lip	31%	48%	52%	0%	0%
II	Soft palate	5%	60%	40%	0%	0%
III	Tongue	32%	26%	7%	67%	7%
IV	Larynx	31%	0%	0%	100%	24%

*ICU,* intensive care unit.
